# IMPACT: A Generative AI Prompt Framework for Scaling Instructional Video Design

**DOI:** 10.1007/s40670-026-02784-7

**Published:** 2026-06-09

**Authors:** Mohit Gupta, Jake Aumeier, Smrithi Upadhyayula, Aidan Dulaney, Alexander Wu, James Willig, Natalie Bavli

**Affiliations:** 1 UT Southwestern Medical Center, Dallas, TX USA; 2 Division of Hematology, Department of Internal Medicine, UT Southwestern Medical Center, 2001 Inwood Road, Dallas, TX 75390 USA

**Keywords:** Generative AI, Prompt engineering, Multimedia learning, Video-based learning

## Abstract

Generative AI can transform lectures into asynchronous learning materials, but pedagogical quality depends on rigorous prompt engineering. We developed IMPACT, a six-component prompt-engineering framework, and applied it to design a six-stage video pipeline. Trainee-faculty co-creation produced 22 pre-clerkship hematology videos and was associated with positive trainee contributor perceptions.

**Supplementary Information:**

The online version contains supplementary material available at https://doi.org/10.1007/s40670-026-02784-7.

Flipped classroom models in pre-clerkship medical education depend on asynchronous materials that prepare students for higher-order in-class learning. However, traditional lectures are not consistently designed using Cognitive Theory of Multimedia Learning (CTML) principles such as segmenting, coherence, and personalization [1, 2]. Generative AI offers a potential solution: large language models (LLMs) are increasingly applied across health professions education for content generation and personalized learning, though their usefulness depends on structured prompts that constrain the model toward accurate and pedagogically aligned outputs [3]. Structured prompts may also lower the barrier for trainees to co-create content alongside faculty, despite limited instructional design experience [4].

We developed IMPACT, a prompting framework comprising six advanced prompt-engineering techniques: Iterative refinement, Meta-prompting, Prompt chaining, Alignment to theory, Contextual exemplars, and Tiered validation. We applied IMPACT to design a six-stage prompt chain that transforms traditional lectures into scripts and slides for instructional videos.

Each technique addresses a distinct aspect of prompt design (Table 1). Iterative refinement evolves the prompts across cycles; meta-prompting directs the model to self-monitor against named failure modes; and prompt chaining decomposes the workflow into sequential, role-specific stages. Alignment to theory encodes established educational theory as prompt rules, while contextual exemplars supply curated reference outputs that calibrate tone and structure. Tiered validation combines in-prompt self-checks and between-stage human review.

Building on foundational prompt-engineering practices, we used IMPACT to design a six-stage prompt chain that transforms traditional lectures into short instructional videos [5]. The chunking prompt segments lecture content into short, objective-aligned videos, each focused on a single cognitive goal. The blueprint prompt establishes the reasoning arc, vignette structure, active learning prompts, and anticipated learner confusions. Drafting and refinement prompts convert this blueprint into tutor-style narration. Slide-generation prompts then convert the finalized narration into slide units and high-yield bullets, after which the deck is assembled using an AI-assisted presentation tool (Gamma; https://gamma.app).

Prompts operationalize CTML principles by dividing lectures into short videos (segmenting), removing extraneous detail (coherence), modeling reasoning through case vignettes (worked examples), prompting learner-generated responses (generative activity), and using conversational language (personalization).

We applied this workflow across five trainee-faculty co-creation workshops, producing 22 short videos for a pre-clerkship hematology course. After each workshop, we administered a newly developed, author-designed reflection survey. All 24 trainee contributors completed Likert and free-response items (100% response rate; Supplementary Table 1). Respondents reported positive perceptions of ownership, meaningful contribution, content design learning, skill development, and emerging educator identity.

IMPACT provided a structured prompt-engineering approach for designing an AI-assisted video-production workflow grounded in CTML principles. In this initial application within a pre-clerkship hematology course, the workflow supported production of 22 short videos and enabled trainee contributors to co-create materials with faculty despite limited prior instructional design experience. Preliminary survey findings suggest that contributors perceived the process as valuable, but these data do not yet demonstrate video effectiveness, improved learner outcomes, production efficiency, or validated quality improvement. Faculty perspectives were not systematically captured. Future work will evaluate faculty experience, development time, content fidelity, learner engagement with the videos, and downstream learning outcomes.

**Table 1:** The IMPACT framework applied to the Video-Based Learning (VBL) pipeline

Letter	Principle	Definition	Application to the VBL pipeline
I	Iterative refinement	A human-in-the-loop prompt improvement process in which model outputs are reviewed, edited to exemplar quality, and used to identify prompt-chain limitations that inform revisions before the next run.	Across iterations, recurring failure modes (e.g., content compression) were diagnosed and encoded as explicit prompt constraints in the next version.
M	Meta-prompting	Instructing the model to watch for and avoid named, recurring failure modes by stating those failure modes explicitly within the prompt.	Early outputs revealed that the model defaulted to summarization instead of step-by-step explanation, a serious failure mode for first-exposure learners. The prompt names this directly (“you tend to summarize rather than explain step-by-step”).
P	Prompt chaining	Decomposing a complex task into a sequence of single-purpose prompts in which each stage’s output becomes the structured input for the next stage.	The pipeline comprises six sequential stages (chunking, blueprinting, drafting, refining, slide segmentation, and bullet conversion), each performing one instructional design job.
A	Alignment to theory	Embedding established learning-science principles directly into prompt instructions so that outputs are pedagogically grounded by default, without requiring authors to be instructional designers.	Each stage encodes specific CTML principles (segmenting, coherence, worked examples, generative activity, and personalization).
C	Contextual exemplars	Curated reference outputs are supplied with each prompt to calibrate tone, structure, level of detail, pacing, and quality threshold.	Each stage embeds 3 to 5 diverse exemplars from prior in-house videos, so the model can learn the instructional arc, pacing, and active-prompt placement.
T	Tiered validation	Pairing automatic checks (in-prompt validation gates and, at high-stakes stages, cross-model verification) with structured human review between stages.	Each stage closes with a checklist the model treats as a gate, and high-stakes stages add cross-model verification (one model drafts, another audits). Output is reviewed and edited by humans between each stage.

## Supplementary Information

Below is the link to the electronic supplementary material.

Supplementary Material 1

Supplementary Material 2

## Declarations

### AI Use Statement

Generative AI tools (ChatGPT-4o and Gemini 1.5 Flash) were used to support prompt development and video-based learning workflow. Gamma (Gamma.app, Inc.) was used to generate initial slides. AI tools assisted with light editing of this manuscript. All AI-generated content was reviewed, edited, and approved by the authors.

### Previous Presentations

Poster presentation at Collaborative Advancement in Research and Medical Education Conference, Dallas, TX, United States. Oct 18, 2025.
