# Supplementary material for: IMPACT: A Generative AI Prompt Framework for Scaling Instructional Video Design
Source: Med Sci Educ. 2026 Jun 9;36(4):1839–41. doi: 10.1007/s40670-026-02784-7 (PMC13582745; doi:10.1007/s40670-026-02784-7)
Supplement: Supplementary file 1 [file 40670_2026_2784_MOESM1_ESM.docx]

**Supplementary Appendix 1. Representative Prompt Excerpts from the IMPACT-Aligned Video Pipeline**

This appendix illustrates how the IMPACT framework is implemented in the prompt chain. The pipeline has continued to evolve since the pilot reported in this manuscript; the verbatim excerpts below are drawn from the current production version (V7.2), which incorporates iterative refinements informed by the pilot. The core IMPACT structure—alignment to theory, meta-prompting, prompt chaining, tiered validation, contextual exemplars, and iterative refinement—is preserved across versions.

**Representative excerpts**

The excerpts below illustrate alignment to theory, meta-prompting, and tiered validation—the IMPACT components whose implementation is most visible in prompt text.

**A — Alignment to theory**

The manuscript identifies Cognitive Theory of Multimedia Learning principles operationalized within the prompt chain (Table 1; paragraph 5). The excerpts below show how these principles were translated into specific prompt instructions across successive stages of the pipeline.

**Segmenting**

Stage 0 prompts the model to divide lecture content into a limited number of short, cognitively coherent video chunks:

*Group the merged content into the minimum number of chunks (3–6). For each candidate chunk, apply the Chunk Justification Test FIRST — does this content earn its own video? Only chunks that pass the Justification Test become final chunks; content that fails it gets absorbed.*

Stage 0 also sets explicit duration boundaries for each chunk:

*A chunk should be teachable in approximately 7–12 minutes once scripted (5–15 min when cognitive load justifies). Prefer the lower end when content allows.*

**Coherence**

Stage 0 prompts the model to remove material that does not serve the preparatory purpose of the video series:

*Cut content that serves neither Step 1 foundations; falls outside the lecture’s topical scope; is beyond Step 1 depth and not required for the case discussion.*

**Worked examples**

Stage 1 prompts the model to anchor the script blueprint in a clinical vignette and return to that vignette at key reasoning moments:

*Anchor in 1 case (default) or 2 cases (if contrast is essential). Revisit at every reasoning pivot: typically 3–5 callbacks in a single-case video.*

Stage 2 then prompts the model to format those callbacks consistently throughout the narrated script:

*Use 3–5 explicit callbacks at Blueprint-designated reasoning pivots. Format explicit callbacks as: “Back to [Patient Name]…”*

**Generative activity**

Stage 2 prompts the model to include active learning prompts that are varied and distributed.

*Across the script: typically 2–4 prompts, varied types, spaced. At least one mid-video reasoning prompt and one at the return to guiding questions.*

The prompt also requires the tutor to debrief each prompt rather than leaving it as an unsupported pause:

*[Spoken prompt — natural tutor voice. Example: “Take a moment — if the marrow were responding normally, what would you expect the reticulocytes to do?”]*

*[Tutor debrief — 1–3 sentences. Example: “Exactly — you’d expect it to rise to compensate. If it’s low, that’s a clue the marrow isn’t mounting a response.”]*

**Personalization**

Stages 1, 2, and 3 share a canonical Voice and Style Card that promotes a conversational tutor voice:

*First person. The teacher narrates as themselves. Open with “Welcome back” / “Hello, everyone — my name is [Teacher Name]” before stating the topic.*

*Conversational connectives. “Let’s…,” “Now,” “So,” “But here’s the twist,” “Let me emphasize that,” “Here’s how I’m thinking about this.”*

*Address the learner directly. “You’ll see,” “Notice that,” “Take a moment and think…”*

**M — Meta-prompting**

Meta-prompting is implemented by naming the failure modes the model is most likely to commit and instructing the model to avoid them. The excerpt below shows representative failure modes the script blueprint stage is instructed to prevent.

- *Undefined acronyms or assumed prior knowledge*
- *Textbook/encyclopedic voice*
- *Summarizing instead of building a mental model step-by-step*

**T — Tiered validation**

Validation is implemented as a three-layer architecture declared explicitly within the prompt: an in-prompt checklist the model treats as a pre-output gate, cross-model verification by an independent model, and human review before downstream use.

Each stage closes with a checklist the model executes before output. For example, the Stage 2 draft script closes with a checklist that includes:

- *3–5 explicit vignette callbacks at reasoning pivots*
- *2–4 active learning prompts, each with debrief*
- *Must define terms before first use*
- *Each segment ends with 1-sentence micro-recap + 1-sentence transition*
- *Tutor voice throughout; pacing unhurried, supportive*
