# Supplementary material for: IMPACT: A Generative AI Prompt Framework for Scaling Instructional Video Design
Source: Med Sci Educ. 2026 Jun 9;36(4):1839–41. doi: 10.1007/s40670-026-02784-7 (PMC13582745; doi:10.1007/s40670-026-02784-7)
Supplement: Supplementary file 2 [file 40670_2026_2784_MOESM2_ESM.docx]

**Supplementary Table 1. Learner Responses to Reflection Survey (N = 24).**

| **Survey Item** | **% Strongly Disagree** | **% Disagree** | **% Neutral** | **% Agree** | **% Strongly Agree** |
| --- | --- | --- | --- | --- | --- |
| Sense of ownership | 0% | 0% | 4% | 46% | 50% |
| Meaningful Contribution | 0% | 0% | 4% | 46% | 50% |
| Content Design Learning | 0% | 0% | 8% | 34% | 58% |
| Ideas Were Valued | 0% | 0% | 4% | 25% | 71% |
| Developed Skills | 0% | 0% | 17% | 25% | 58% |
| Developed Educator Identity | 0% | 0% | 8% | 34% | 58% |
